# Supplementary figures and images for: New insights in the allelopathic traits of different barley genotypes: Middle Eastern and Tibetan wild-relative accessions vs. cultivated modern barley
Source: PLoS One. 2020 Apr 23;15(4):e0231976. doi: 10.1371/journal.pone.0231976 (PMC7179892; doi:10.1371/journal.pone.0231976)

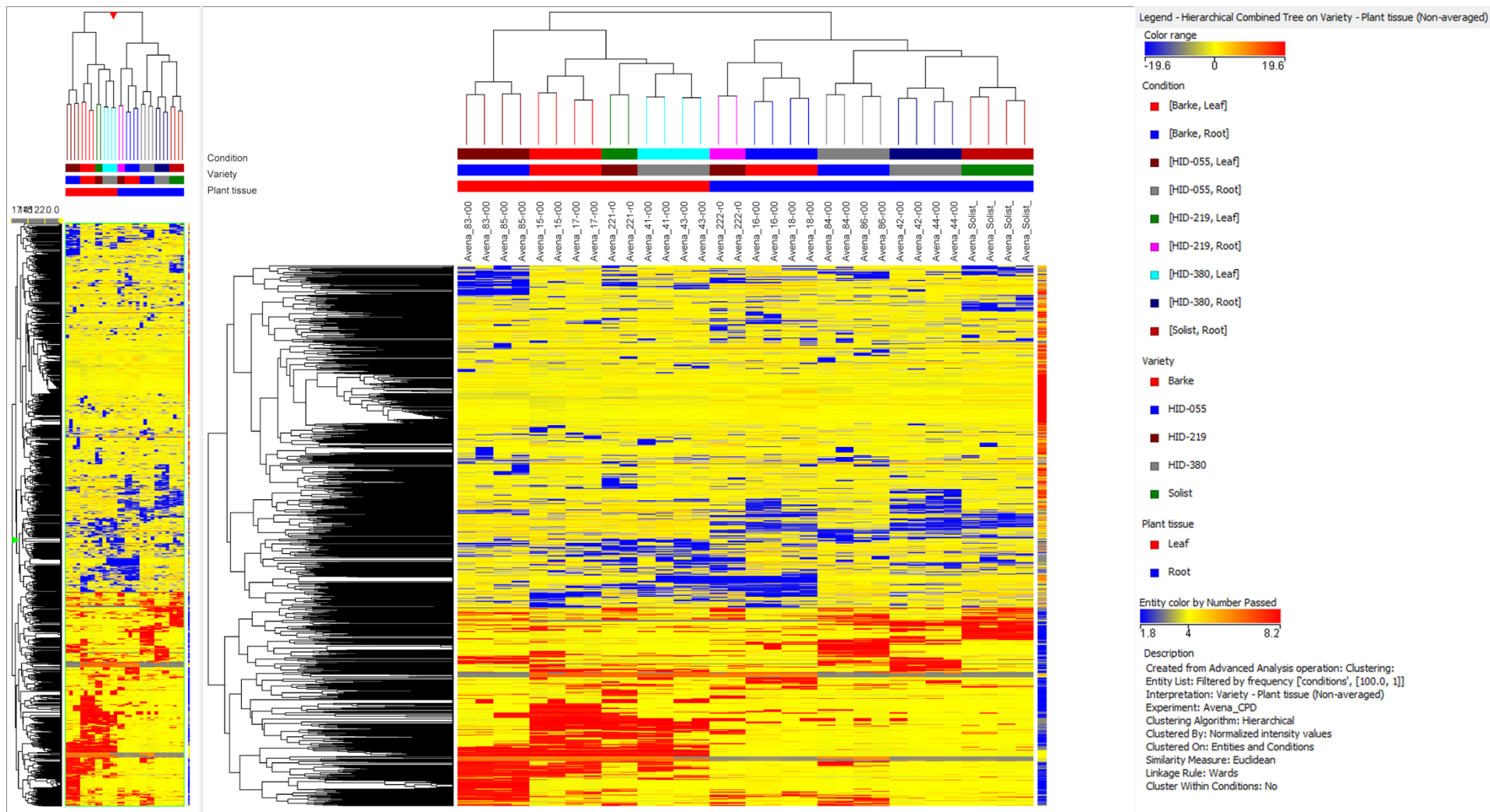

Supplement: S1 Fig — Unsupervised hierarchical cluster analysis from the fold-change based heatmaps in leaves and roots of wild relatives and modern cultivated barley. (PDF) [file pone.0231976.s001.pdf]
