# Supplementary material for: New insights in the allelopathic traits of different barley genotypes: Middle Eastern and Tibetan wild-relative accessions vs. cultivated modern barley
Source: PLoS One. 2020 Apr 23;15(4):e0231976. doi: 10.1371/journal.pone.0231976 (PMC7179892; doi:10.1371/journal.pone.0231976)
Supplement: S1 Table — Barley accessions by origin group: T-NS = Turkey near Diyarbakir and northern Syria, Iq-In = Northern Iraq and western Iraq, T-G = Turkey near Gaza, L-WS = Lebanon-western Syria, In = Southwestern Iran, Asia = Central Asia, I-J = Israel-Jordan, H.ag = Tibet. GS = Genetic similarity between donor and Barke, based on simple matching. (PDF) [file pone.0231976.s002.pdf]

Donor accession	Origin	Origin Group	GS to Barke
HID-003	Iraq	Iq-In	0.54
HID-004	Iraq	Iq-In	0.45
HID-055	Turkey	T-NS	0.46
HID-062	Turkey	T-G	0.48
HID-065	Turkey	T-G	0.44
HID-099	Syria	L-WS	0.46
HID-101	Syria	L-WS	0.46
HID-102	Syria	L-WS	0.47
HID-109	Syria	L-WS	0.46
HID-114	Lebanon	L-WS	0.46
HID-138	Iran	In	0.45
HID-140	Iraq	Iq-In	0.46
HID-144	Iran	In	0.46
HID-219	Afghanistan	Asia	0.46
HID-294	Iran	In	0.47
HID-295	Iran	T-NS	0.40
HID-357	Turkey	T-NS	0.45
HID-358	Turkey	T-NS	0.45
HID-359	Israel	I-J	0.46
HID-380	China	H.ag (Tibet)	0.45
HID386	Israel	I-J	0.46
Barke	Germany	-	1.00
