# Supplementary material for: New insights in the allelopathic traits of different barley genotypes: Middle Eastern and Tibetan wild-relative accessions vs. cultivated modern barley
Source: PLoS One. 2020 Apr 23;15(4):e0231976. doi: 10.1371/journal.pone.0231976 (PMC7179892; doi:10.1371/journal.pone.0231976)
Supplement: S2 Table — Discriminant phenolic compounds identified by VIP (Variable Importance in Projection) analysis following OPLS-DA discriminant analysis in Barley leaves. Compounds are provided together with VIP scores (measure of variable’s importance in the OPLS-DA model) > 1.6. (PDF) [file pone.0231976.s003.pdf]

Class	Compound	VIP Score	Fold-change Modern vs ancient
**Alkaloids and derivatives**	(R)-N-methylcoclaurine	1.75084	3.92
	(S)-N-methylcoclaurine// codeine	1.76882	2.92
	cathinone	1.72991	1.68
	gramine	1.66464	-6.17
	methylnicotinate	1.60727	0.98
**Hormones**	16, 17-dihydro-16α;, 17-dihydroxy gibberellin A_9	1.64563	0.83
	16,17-dihydro-16α,17-dihydroxy gibberellin A_{12}	1.69372	2.98
	3-hydroxy-2-oxo-(indol-3-yl)acetate	1.61908	0.05
	7-hydroxy-2-oxindole-3-acetate	1.6084	1.32
	benzyladenine-N-glucoside	1.75241	5.22
	dihydrozeatin-9-N-glucoside-O-glucoside	1.70651	1.31
	dioxindole-3-acetyl-3-O-β-glucose	1.75865	1.67
	gibberellin A_{44} (closed lactone form)	1.60361	0.65
	indole-3-acetyl-methionine	1.81683	18.33
**Phenolic compounds**	(-)-yatein	1.75774	1.55
	(S)-colchicine	1.68329	1.50
	5-O-caffeoylshikimate	1.68429	0.55
	6-C-glucosyl chrysin	1.68081	1.15
	cyanidin 3-O-(6-O-β-D-glucosyl-2-O-β-D-xylosyl-β-D-galactoside)// cyanidin 5-O-β-D-glucoside 3-O-β-D-sambubioside	1.84388	18.44
	cyanidin 3-O-β-D-caffeoylglucoside	1.75829	0.79
	cyanidin 3-O-β-D-sambubioside	1.75747	0.59
	dalpatein 7-O-β-D-apiofuranosyl-(1->6)-β-D-glucopyranoside	1.73921	0.39
	delphinidin 3,5-di-O-β-D-glucoside// delphinidin 3-O-sophoroside// quercetin 3,4'-O-diglucoside// delphinidin 3,7-di-O-β-D-glucoside// quercetin 3-O-sophoroside// quercetin-3-gentiobioside// quercetin A-O-diglucoside// quercetin 3-O-β-D-glucosylgalactoside	1.70699	1.30
	dihydroconiferyl alcohol	1.74528	2.01

	isorhamnetin 3-O-(6''-O-feruloyl)-glucoside	1.86575	16.60
	isorhamnetin 3-O-(N-O-p-coumaroyl)-glucoside// kaempferol 3-O-(6\-O-feruloyl)-glucoside''	1.67972	2.26
	isovitexin 2''-O-rhamnoside// vitexin 2''-O-β-L-rhamnoside// pelargonidin 3-O-β-D-p-coumaroylglucoside	1.75495	0.54
	isovitexin 2''-O-β-D-glucoside	1.74837	0.45
	isovitexin-7-O-glucosyl-2''O-rhamnoside	1.83728	2.16
	kaempferol 3-O-(N-O-di-p-coumaroyl)-glucoside''// pelargonidin-3,5-di-O-β-D-glucoside// isovitexin 7-O-glucoside// pelargonidin 3-O-sophoroside// genistin 7-gentiobioside// apigenin-7-O-gentiobioside// pelargonidin 3,7-di-O-β-D-glucoside// isovitexin 7-O-galactoside// luteolin 7-O-neohesperidoside// kaempferol 3-O-rhamnoside-7-O-glucoside// 4-(2-aminophenyl)-2,4-dioxobutanoate// cyanidin-3-O-rutinoside	1.61968	0.50
	kaempferol-3-glucoside-7-rhamnoside	1.75105	0.48
	maysin	1.74213	0.61
	pelargonidin-3-O-rutinoside-5-O-β-D-glucoside	1.7845	1.62
	peonidin 3,5-diglucoside	1.67952	2.33
	quercetin 3-O-(4''-O-p-coumaroyl)-glucoside	1.76631	2.51
	quercetin 3-O-(N-O-p-coumaroyl)-glucoside	1.77225	2.28
	quercetin-3-rhamnoside-7-rhamnoside// vicenin-2// vitexin 2''-O-β-D-glucoside// rhamnosylisoorientin	1.86977	24.63
**Serotonin amide**	cinnamoylserotonin	1.75397	3.14
	N-acetyl-serotonin	1.79729	19.58
**Terpenoids**	9α-copalyl diphosphate	1.61702	0.65
	iridotrial	1.75957	0.58
	steviolbioside// rubusoside	1.72091	1.25
**Others**	(+)-copalyl diphosphate// 2-cis,6-trans,10-trans-geranylgeranyl diphosphate// geranylgeranyl diphosphate// (13E)-labda-7,13-dien-15-yl diphosphate// (-)-ent-copalyl diphosphate	1.62715	0.25
	(R)-prunasin	1.79616	0.67
	1-[18-hydroxyoleyl]-2-lyso-phosphatidate	1.79751	1.76
	1-O-4-hydroxybenzoyl-β-D-glucose	1.7962	0.71

3,5-di-C-glucosyl-2,4,4',6-tetrahydroxydibenzoylmethane	1.78858	-3.46
3-cyano-L-alanine	1.61786	-11.33
3-ureidopropanoate	1.61747	-9.45
4-(1-methyl-2-pyrrolidinyl)-3-oxobutanoate methyl ester	1.87754	20.28
7-hydroxy-2-oxindole-3-acetate glucoside	1.76168	1.79
7-methylinosine	1.69694	1.51
adipate	1.6134	1.29
allocryptopine	1.82529	2.57
benzoyl-β-D-glucopyranose	1.66065	1.55
bisorganyltrisulfane	1.86579	16.87
chorismate/isochorismate	1.61097	-0.12
diethylpyrocarbonate	1.85959	1.68
ferric biliverdine-IX-α	1.73224	0.35
glutathione disulfide	1.76599	2.28
homoglutathione	1.61281	0.92
indole-3-carboxylate	1.68643	0.53
L-galactono-1,4-lactone// 3-keto-β-D-galactose// L-sorbosone// D-glucono-1,5-lactone// L-gulono-1,4-lactone// L-sorbosone-N-lactone	1.84421	17.19
phenylethyl acetate// eugenol// 4-hydroxyphenylbutan-2-one//	1.7739	0.63
S-(2-aminoethyl)-L-cysteine	1.66501	-1.18
S-methylglutathione	1.61281	0.92
xylogalacturonan	1.70485	0.51
α-hydroxyheme	1.7271	17.56
