# Supplementary material for: New insights in the allelopathic traits of different barley genotypes: Middle Eastern and Tibetan wild-relative accessions vs. cultivated modern barley
Source: PLoS One. 2020 Apr 23;15(4):e0231976. doi: 10.1371/journal.pone.0231976 (PMC7179892; doi:10.1371/journal.pone.0231976)
Supplement: S3 Table — Discriminant phenolic compounds identified by VIP (Variable Importance in Projection) analysis following OPLS-DA discriminant analysis in Barley roots. Compounds are provided together with VIP scores (measure of variable’s importance in the OPLS-DA model) > 1.6. (PDF) [file pone.0231976.s004.pdf]

Class	Compound	VIP Score	Fold-change Modern vs ancient
**Alkaloids**	4,21-dehydrogeissoschizine	1.71177	-1.17
	betanidin quinone	1.82021	17.06
	cephaeline	1.6907	-2.17
	geissoschizine	1.60554	-19.15
	lupinate	1.60862	2.11
	norephedrine// norpseudoephedrine	1.71129	-0.34
	raucaffricine	1.61503	-0.66
**Hormones**	24-epibrassinolide// brassinolide	1.6306	-20.64
	gibberellin A_{29}-catabolite	1.82296	2.94
**Phenolic compounds**	(-)-4'-demethyl-deoxypodophyllotoxin// S-adenosyl-L-homocysteine// 3-(carbamoylamino)-2-methylpropanoate	1.60228	2.27
	(-)-epicatechin-3-O-gallate	1.84802	17.12
	(-)-vestitol	1.77714	-1.02
	(2S)-pinocembrin	1.69125	-2.40
	eugenol// 4-hydroxyphenylbutan-2-one// propyl benzoate// pseudoisoeugenol	1.90362	3.07
	justicidin B	1.61726	2.09
	olivetol	1.79503	4.06
**Secondary metabolites**	3-epihydroxy-2'-deoxymugineate	1.74653	-3.69
	TRIBOA-β-D-glucoside	1.61835	1.72
**Terpenoids**	(2Z,6Z)-farnesyl diphosphate	1.81648	17.05
	iridotrial// coniferyl alcohol	1.90146	3.29
**Others**	(6R)-4a-hydroxy-tetrahydrobiopterin	1.70629	1.57
	(E)-1-(L-cysteinylglycin-S-yl)-N-hydroxy-ω-(methylsulfanyl)nonan-1-imine	1.75344	1.11
	1,3-dioctanoylglycerol	1.65408	0.49
	10-methyl-5,6,7,8-tetrahydropteroylglutamate	1.65946	1.67
	2,5-dihydroxybenzoate A-O-β-D-glucoside	1.67834	18.47

2-[(2'-methylthio)hexyl]maleate	1.65401	0.45
3-methylbutanal// 3-methyl-2-butanone// (E)-2-pentenol// 1-pentan-3-one// isoprenyl alcohol	1.67956	-0.12
3-methylxanthosine	1.80884	0.75
5'-(p-nitrophenyl)thioadenosine	1.78488	17.73
5-azacytidine	1.62757	-17.06
7-methylxanthine	1.61769	0.99
8-aminoethyl-2,6-anhydro-3,8-dideoxy-D-glycero-D-talo-octonate// N-acetyl-β-D-glucosaminyl group	1.77752	-17.82
choline	1.67713	-0.11
conhydrine// pseudo-conhydrine// 8-aminooctan-4-one	1.65411	3.37
cumene hydroperoxide	1.71245	-0.34
ecgonine methyl ester// 4-(1-methyl-2-pyrrolidinyl)-3-oxobutanoate methyl ester	1.89769	2.42
hydratedusnate// 1,4,5-trihydroxy-6,7,8-trimethoxy-2-methylanthraquinone// a trimethylmyricetin// a trimethylquercetagetin	1.61961	1.74
L-cycloserine	1.60986	0.44
lesquerolate	1.78831	-0.22
N,N-dihydroxypentahomomethionine	1.61753	-1.13
N^4-aminocytidine	1.65326	1.37
N-dimethylethanolamine phosphate	1.6011	2.81
O-sinapoylglucarolactone	1.61112	1.61
O-ureido-L-homoserine	1.78204	19.06
pantetheine	1.81473	-1.16
phenanthrene-ring	1.74122	-0.22
phenylacetaldehyde	1.84451	1.64
propionyl adenylate	1.85081	18.51
salidroside	1.62128	-1.49
thymine	1.65954	1.93
uracil	1.6596	-1.34
