# Supplementary material for: New insights in the allelopathic traits of different barley genotypes: Middle Eastern and Tibetan wild-relative accessions vs. cultivated modern barley
Source: PLoS One. 2020 Apr 23;15(4):e0231976. doi: 10.1371/journal.pone.0231976 (PMC7179892; doi:10.1371/journal.pone.0231976)
Supplement: Attachment — Submitted filename: Response to Reviewers.docx [file pone.0231976.s005.docx]

Dear Editor and referees,

We have appreciated the observations you have raised and we think that, thanks to your suggestions and advice, we could further improve the quality of our work.

As follows, a point-to-point response to the criticisms raised has been provided. The changings to the text have been added in the “Revised Manuscript with Track Changes” file.

Best regards,

Mauro Maver

**Reviewer # 1:**

1. The author should reconstruct the “abstract” logically to make it easy for reading, specifically from line 45-52. And it is better to add one conclusion sentence at the end of the abstract

*We amended the abstract, also adding a conclusion sentence, making the abstract easier to read.*

2. Line236-238 the figure legend should be before the figure not in the text. The layout should be adjusted to make it good to read.

*This has been changed accordingly.*

3. The purpose of this investigate is to use allelochemicals for weeding, so why do you choose lettuce to test the phytotoxic? In current situation, lettuce is not a kind of weed growing in crop field, right?

*Thanks for the comment. Yes, lettuce is not a weed in crop fields but at the same time, lettuce has been well known and demonstrated as a sensitive plant to phytotoxic substances, and it has been widely used in phytotoxic investigations. Typical plant bioassay species used to investigate ecotoxicity of chemicals and soil amendments to higher terrestrial plants include lettuce: ASTM, 2003; ISO, 2005; OECD/OCDE, 2006a, b*

- *ASTM (American Society for Testing and Materials), 2003. ASTM E1963 – 02: Standard guide for conducting terrestrial plant toxicity tests. American Society for Testing and Materials, Philadelphia, PA, Philadelphia, PA.*
- *ISO (International Organization for Standardization), 1993. ISO 11269-1. Soil quality: 828 determination of the pollutants 1 effects on soil flora. Part 1. Method for the measurement of inhibition of root growth. International Organization for Standardization, Geneva.*
- *ISO (International Organization for Standardization), 2005. ISO 11269-2. Soil quality: 831 determination of the pollutants effects on soil flora. Part 2. Effects of chemicals on the emergence and growth of higher plants. International Organization for Standardization, Geneva.*

4. Line 293, in group, should be lower case.

*We added the article “the” to specify the In group and to avoid further misunderstanding.*

**Reviewer # 2:**

1. NAM is short for nested associated mapping, authors don’t need to put the full name before the abbreviations.

*This has been changed accordingly.*

2. Line 93, “have been observed that…”, who?

*This has been changed accordingly..*

3. Line 381 “ in the early 50ies”. Please revise them.

*This has been changed accordingly.*
